# Supplementary material for: Effect size estimates from umbrella designs: Handling patients with a positive test result for multiple biomarkers using random or pragmatic subtrial allocation
Source: PLoS One. 2020 Aug 14;15(8):e0237441. doi: 10.1371/journal.pone.0237441 (PMC7428134; doi:10.1371/journal.pone.0237441)
Supplement: S1 File — Code to reproduce the results of the analytical calculations and of the simulation study of this article. (ZIP) [file pone.0237441.s001.zip › _README.pdf]

README for the R source code

belonging to the manuscript "*Effect size estimates from umbrella designs: handling patients with a positive test result for multiple biomarkers using random or pragmatic subtrial allocation*" by Miriam Kesselmeier, Norbert Benda and André Scherag

Author of the source code:

Miriam Kesselmeier (miriam.kesselmeier@med.uni-jena.de)

Content:

The files provide R source code to re-run the analyses (except for the real data application) presented in the corresponding manuscript.

Table of Contents:

Introduction 2

Computation time and R session information 2

Illustration of the analytical derivations 2

Simulation study 2

Instructions to apply the R source code 3

Illustration of the analytical derivations 3

Simulation study 3

Introduction

This is the README for the R source code that can be used to re-run both the analytical derivations and the simulation study. The provided code produces the figures and the numbers provided in the manuscript. We provide in this README the computation time for each analysis and the corresponding R session information (first part of the README). In the second part, we provide file descriptions as well as instructions to re-run the analyses.

Here, we term the difference between the estimated treatment effects in the independent trial design and a corresponding umbrella subtrial bias.

Computation time and R session information

Illustration of the analytical derivations

Executed file: Run_AnalyticalCalculations.R

- computation time: within seconds
- R session information:

  R version 3.5.1 (2018-07-02)
  Platform: x86_64-w64-mingw32/x64 (64-bit)
  Running under: Windows 7 x64 (build 7601) Service Pack 1

  Matrix products: default

  locale:
  [1] LC_COLLATE=German_Germany.1252 LC_CTYPE=German_Germany.1252
  LC_MONETARY=German_Germany.1252 LC_NUMERIC=C
  LC_TIME=German_Germany.1252

  attached base packages:
  [1] stats   graphics grDevices utils   datasets methods   base

  other attached packages:
  [1] zipfR_0.6-66

  loaded via a namespace (and not attached):
  [1] compiler_3.5.1 tools_3.5.1

Simulation study

Session information

R version 3.5.1 (2018-07-02)
Platform: x86_64-redhat-linux-gnu (64-bit)
Running under: CentOS Linux 7 (Core)
Matrix products: default
BLAS/LAPACK: /usr/lib64/R/lib/libRblas.so

locale:
[1] LC_CTYPE=en_US.UTF-8   LC_NUMERIC=C
[3] LC_TIME=en_US.UTF-8   LC_COLLATE=en_US.UTF-8
[5] LC_MONETARY=en_US.UTF-8   LC_MESSAGES=en_US.UTF-8
[7] LC_PAPER=en_US.UTF-8   LC_NAME=C
[9] LC_ADDRESS=C   LC_TELEPHONE=C
[11] LC_MEASUREMENT=en_US.UTF-8 LC_IDENTIFICATION=C

attached base packages:

[1] stats graphics grDevices utils datasets methods base
loaded via a namespace (and not attached):
[1] compiler_3.5.1

Computation time for file execution

- BiomarkerStatusAdmixtureSimulationbased_Example.R: approx. 6.3 h
- BiomarkerStatusAdmixtureSimulationbased_Example2.R: approx. 3.9 h
- Simulation_Example.R: approx. 10 min

Instructions to apply the R source code

Illustration of the analytical derivations

The code can be applied for (i) different allocation ratios r for patients with a positive test result for both biomarkers and for (ii) dependent biomarkers with dependency measure ϕ without additional effort.

File information:

- Files with function definitions:
  - Functions_AnalyticalCalculations.R
- Files to be executed:
  - Run_AnalyticalCalculations.R

Execution:

1. Run Run_AnalyticalCalculations.R to get all numbers and plots.

Results:

- Fig_Analytical_Recruitmenttime.png corresponds to figure 2 in the article
- Fig_Analytical_Biomarkerstatusdistribution.png corresponds to figure 3 in the article
- Fig_Analytical_Prob_Closing.png corresponds to figure 4 in the article
- Fig_Analytical_Bias_h1.png corresponds to figure 5 in the article

Simulation study

This code can easily be modified to run it with different biomarkers. If one wants to apply the pragmatic allocation scheme in an umbrella design for a low prevalence, the number of simulation runs must be increased for the simulation-based estimation of the expected biomarker status distribution.

File information:

- Files with function definitions:
  - Functions_AnalyticalCalculations.R contains the definitions of those functions that are needed to calculate the expected biomarker status-related true treatment effect.
  - FunctionBiomarkerStatusAdmixtureSimulationbased.R contains the definitions of those functions that are needed to estimate the expected biomarker status distribution based on simulations.
  - Functions_for_Simulation.R contains the functions to simulate the (sub-) trials and to subsequently analyse the simulated (sub-) trials. The functions are ordered chronologically.
- Files to be executed:
  - Comparison of the subtrial allocation schemes:
    - BiomarkerStatusAdmixtureSimulationbased_Example.R contains the source code to estimate the expected biomarker status distribution based on simulations.
  - Comparison of the subtrial analysis methods:

  - BiomarkerStatusAdmixtureSimulationbased_Example2.R contains the source code to estimate the expected biomarker status distribution based on simulations.
  - Simulation_Example.R contains the source code to run the main simulation study and provides the calculation of the performance measures.
  - Plots_Simulation.R contains the source code to reproduce the plots.

Execution:

- Comparison of the subtrial allocation schemes:
  1. Estimate simulation based the expected biomarker status distribution, i.e. run BiomarkerStatusAdmixtureSimulationbased_Example.R.
- Comparison of the subtrial analysis methods:
  1. Estimate simulation based the expected biomarker status distribution, i.e. run BiomarkerStatusAdmixtureSimulationbased_Example2.R.
  2. Run the simulation study, i.e. run Simulation_Example.R.
  3. Plot the results, i.e. run Plots_Simulation.R.

Results:

- Comparison of the subtrial allocation schemes – numbers given in the manuscript are extracted from the following files:
  - Recruitment time (number of discarded patients):
    i. Discard_Umbrelladesign_Random_anlauf3.txt contains the number for the umbrella design with the random allocation scheme.
    ii. Discard_Umbrelladesign_SmallerStudy_anlauf3.txt contains the number for the umbrella design with the pragmatic allocation scheme
    iii. Discard_Paralleldesign_anlauf3.txt contains the number for the independent trial design
  - Biomarker status distribution:
    i. Admixture_Umbrelladesign_Random_anlauf3.txt contains the distribution for the umbrella design with the random allocation scheme.
    ii. Admixture_Umbrelladesign_SmallerStudy_anlauf3.txt contains the distribution for the umbrella design with the pragmatic allocation scheme.
    iii. Admixture_Paralleldesign_anlauf3.txt contains the distribution for the independent trial design.
- Comparison of the subtrial analysis methods:
  - Plot_ Bias_Simulation.png corresponds to figure 6 in the article
