# Supplementary material for: Effect size estimates from umbrella designs: Handling patients with a positive test result for multiple biomarkers using random or pragmatic subtrial allocation
Source: PLoS One. 2020 Aug 14;15(8):e0237441. doi: 10.1371/journal.pone.0237441 (PMC7428134; doi:10.1371/journal.pone.0237441)
Supplement: S1 Note — (PDF) [file pone.0237441.s002.pdf]

S1 **Note Umbrella trial design with the random allocation scheme: number of patients needed to be screened and biomarker status distribution**

Settings and notations. Suppose that we have two experimental treatments related to two different, binary biomarkers B_i $(i = 1, 2)$. Let π_i be the prevalence of a positive test result for B_i. We denote the patient's combined test results for all biomarkers the patient's biomarker status. Let ${\mathrm{B}_i}^+$ be an indicator for a positive test result for B_i. Then, let the tuple $(\mathrm{B}_1^+, \mathrm{B}_2^+)$ denote the biomarker (positive) status of a patient. We consider an umbrella trial with two parallel group subtrials and the random subtrial allocation scheme. It is assumed that subtrial i recruits until it has reached the planned sample size N_i. The umbrella trial closes when the second subtrial closes. For further details on the umbrella trial design we refer to the Subsection "Umbrella trial design".

As a dependency between biomarkers might be indicated, we consider the ϕ coefficient as correlation (dependency) measure that is defined as

$$\phi = \frac{\mathrm{P}[(1,1)]\,\mathrm{P}[(0,0)] - \mathrm{P}[(1,0)]\,\mathrm{P}[(0,1)]}{\sqrt{\pi_1\,(1-\pi_1)\,\pi_2\,(1-\pi_2)}} \tag{S1}$$

$\mathrm{P}[(\mathrm{B}_1^+, \mathrm{B}_2^+)]$ denotes the probability for an individual in the disease population to exhibit the biomarker status $(\mathrm{B}_1^+, \mathrm{B}_2^+)$. ϕ is translated into the biomarker status distribution in

the disease population for given π_1, π_2 and ϕ as

$$\begin{aligned} \mathrm{P}[(1,1)] &= \pi_1\,\pi_2 + \phi\,\sqrt{\pi_1\,(1-\pi_1)\,\pi_2\,(1-\pi_2)} \\ \mathrm{P}[(1,0)] &= \pi_1 - \mathrm{P}[(1,1)] \\ \mathrm{P}[(0,1)] &= \pi_2 - \mathrm{P}[(1,1)] \\ \mathrm{P}[(0,0)] &= 1 - \pi_1 - \pi_2 + \mathrm{P}[(1,1)] \end{aligned} \tag{S2}$$

Subtrial allocation of patients. In an umbrella trial, one must distinguish between the time segment where both subtrials are running and the segment where one subtrial is already closed. These segments differ in the proportion of patients with positive test results for both biomarkers that are allocated to the respective subtrial (half versus all). The probability that a patient is allocated to subtrial 1 while both subtrials are under recruitment equals

$$\begin{aligned} p^{(1)}_{0,1} : &\overset{(S2)}{=} (\pi_1 - \mathrm{P}[(1,1)]) + r\,\mathrm{P}[(1,1)] \\ &= \pi_1 - (1-r)\,\mathrm{P}[(1,1)] \end{aligned} \tag{S3}$$

and, analogously, the probability for an allocation to subtrial 2 equals

$$p^{(1)}_{0,2} := \pi_2 - r\,\mathrm{P}[(1,1)] \tag{S4}$$

with the proportion r ($r \in [0,1]$) of patients with a positive test result for both biomarkers that are allocated to subtrial 1 (here: $r = 0.5$). If subtrial 2 is already closed, the probability that a patient is allocated to subtrial 1 is

$$\begin{aligned} p^{(2)}_{0,1} : &\overset{(S2)}{=} (\pi_1 - \mathrm{P}[(1,1)]) + \mathrm{P}[(1,1)] \\ &= \pi_1 \end{aligned} \tag{S5}$$

and, analogously in case subtrial 1 is already closed, the probability for an allocation to subtrial 2 is

$$p^{(2)}_{0,2} := \pi_2 \tag{S6}$$

Number of patients needed to be screened. Consider only patients that are

eligible for the umbrella trial, i.e. patients with a positive test result for at least one of the two biomarkers. The probability that a patient is eligible is given by

$$q_0 := 1 - \mathrm{P}[(0,0)] \tag{S7}$$

First, consider the time segment where patients are recruited for both subtrials. There, the probability that an eligible patient is included in subtrial 1 equals

$$\begin{aligned} p_1^{(1)} : &= \frac{p_{0,1}^{(1)}}{p_{0,1}^{(1)} + p_{0,2}^{(1)}} \\ &\overset{(S3),(S7)}{=} \frac{\pi_1 - (1-r)\,\mathrm{P}[(1,1)]}{q_0} \end{aligned} \tag{S8}$$

The probability for an inclusion in subtrial 2 equals

$$p_2^{(1)} := 1 - p_1^{(1)} \tag{S9}$$

Let X be the number of patients that are included in subtrial 2 when the N_1th patient is included in subtrial 1. Let Y be the number of patients that are included in subtrial 1 when the N_2th patient is included in subtrial 2. Then, X and Y are negative binomial distributed with

$$\begin{aligned} X &\sim \mathrm{NB}(p_1^{(1)}, N_1) \\ Y &\sim \mathrm{NB}(1 - p_1^{(1)}, N_2) \end{aligned} \tag{S10}$$

The probability that subtrial 1 closes first equals the probability that X is smaller than N_2. Then,

$$\begin{aligned} \mathrm{P}[X < N_2] &= \mathrm{P}[X \le N_2] - \mathrm{P}[X = N_2] \\ &= \mathrm{I}_{p_1^{(1)}}(N_1, N_2 + 1) - \mathrm{P}[X = N_2] \\ &= \mathrm{I}_{p_1^{(1)}}(N_1, N_2) \end{aligned} \tag{S11}$$

with the regularized incomplete beta function $\mathrm{I}_{p_1^{(1)}}(\cdot,\cdot)$. The second equality holds as X

follows a negative binomial distribution. The last equality holds because

$$\begin{aligned}
&\mathrm{I}_{p_1^{(1)}}(N_1, N_2+1) \\
&\quad \overset{*_1}{=} \mathrm{I}_{p_1^{(1)}}(N_1, N_2) + \frac{\left(p_1^{(1)}\right)^{N_1}\left(1-p_1^{(1)}\right)^{N_2}}{N_2\, B(N_1, N_2)} \\
&\quad \overset{*_2}{=} \mathrm{I}_{p_1^{(1)}}(N_1, N_2) + \left[N_2 \frac{(N_1-1)!\,(N_2-1)!}{(N_1+N_2-1)!}\right]^{-1} \left(p_1^{(1)}\right)^{N_1}\left(1-p_1^{(1)}\right)^{N_2} \\
&\quad \overset{*_3}{=} \mathrm{I}_{p_1^{(1)}}(N_1, N_2) + \left[\frac{(N_1-1)!\,(N_2)!}{(N_1+N_2-1)!}\right]^{-1} \left(p_1^{(1)}\right)^{N_1}\left(1-p_1^{(1)}\right)^{N_2} \\
&\quad \overset{*_4}{=} \mathrm{I}_{p_1^{(1)}}(N_1, N_2) + \binom{N_2+N_1-1}{N_2} \left(p_1^{(1)}\right)^{N_1}\left(1-p_1^{(1)}\right)^{N_2} \\
&\quad \overset{*_5}{=} \mathrm{I}_{p_1^{(1)}}(N_1, N_2) + \mathrm{P}[X = N_2]
\end{aligned} \tag{S12}$$

with the beta function $B(\cdot,\cdot)$ as well as the application of properties of the regularized incomplete beta function ($*_1$), the definition of the beta function ($*_2$), properties of the factorial ($*_3$), the definition of the binomial coefficient ($*_4$) and properties of the negative binomial distribution ($*_5$). Analogously, the probability that subtrial 2 closes first equals the probability that Y is smaller than N_1. Then,

$$\begin{aligned}
\mathrm{P}[Y < N_1] &= \mathrm{I}_{1-p_1^{(1)}}(N_2, N_1) \\
&= 1 - \mathrm{I}_{p_1^{(1)}}(N_1, N_2)
\end{aligned} \tag{S13}$$

Including the previous deductions, one can derive the expected number of patients that are included in the trial during the time segment where both subtrials are running. In case of subtrial 1 closing first, this number is given by

$$\begin{aligned}
\mathrm{E}_1^{(1)} :&= \mathrm{E}[X|X < N_2] + N_1 \\
&= \left(1 + \frac{1-p_1^{(1)}}{p_1^{(1)}}\, \frac{\mathrm{I}_{p_1^{(1)}}(N_1+1, N_2-1)}{\mathrm{I}_{p_1^{(1)}}(N_1, N_2)}\right) N_1
\end{aligned} \tag{S14}$$

with the conditional expectation $\mathrm{E}[X|X < N_2]$, i.e. the expectation of X given X is

smaller than N_2. The last equality holds because

$$\begin{aligned}
&\mathrm{E}[X|X<N_2]\,\mathrm{P}[X<N_2] \\
&\quad = \mathrm{E}[X\,1_{X<N_2}] \\
&\quad = \sum_{x=0}^{N_2-1} x\,\mathrm{P}[X=x] \\
&\quad = \sum_{x=0}^{N_2-1} x\binom{x+N_1-1}{x}\left(p_1^{(1)}\right)^{N_1}\left(1-p_1^{(1)}\right)^{x} \\
&\quad = \sum_{x=0}^{N_2-1} x\frac{(x+N_1-1)!}{x!(N_1-1)!}\left(p_1^{(1)}\right)^{N_1}\left(1-p_1^{(1)}\right)^{x} \\
&\quad = \sum_{x=1}^{N_2-1} \frac{(x+N_1-1)!}{(x-1)!(N_1-1)!}\left(p_1^{(1)}\right)^{N_1}\left(1-p_1^{(1)}\right)^{x} \qquad \text{(S15)} \\
&\quad = \sum_{x=0}^{N_2-2} \frac{((x+1)+N_1-1)!}{((x+1)-1)!(N_1-1)!}\left(p_1^{(1)}\right)^{N_1}\left(1-p_1^{(1)}\right)^{x+1} \\
&\quad = N_1\sum_{x=0}^{N_2-2} \frac{(x+(N_1+1)-1)!}{x!((N_1+1)-1)!}\left(p_1^{(1)}\right)^{N_1}\left(1-p_1^{(1)}\right)^{x+1} \\
&\quad = \frac{1-p_1^{(1)}}{p_1^{(1)}}\,N_1\sum_{x=0}^{N_2-2}\binom{x+(N_1+1)-1}{x}\left(p_1^{(1)}\right)^{N_1+1}\left(1-p_1^{(1)}\right)^{x} \\
&\quad = \frac{1-p_1^{(1)}}{p_1^{(1)}}\,N_1\,\mathrm{I}_{p_1^{(1)}}(N_1+1,N_2-1)
\end{aligned}$$

with the indicator function $1_{X<N_2}$ (1 if $X<N_2$, 0 otherwise) as well as some calculus with (conditional) expectations, the negative binomial distribution, the regularized incomplete beta function, the binomial coefficient, the factorial, the summation index and multiplication by 1. Now, the last equality in Equation (S14) follows with Equation (S11). Analogously with Equation (S13) in case of subtrial 2 closing first, the expected number of patients that are included in the trial during the time segment where both subtrials are running is given by

$$\mathrm{E}_2^{(1)} := \left(1+\frac{p_1^{(1)}}{1-p_1^{(1)}}\,\frac{\mathrm{I}_{1-p_1^{(1)}}(N_2+1,N_1-1)}{\mathrm{I}_{1-p_1^{(1)}}(N_2,N_1)}\right)N_2 \qquad \text{(S16)}$$

Now, consider the time segment where one subtrial is already closed. Let Z_1 be the number of patients that exhibit a positive test result for at least one biomarker if subtrial 1 closed first. Let Z_2 be analogously defined in case subtrial 2 closed first. Then, it

holds for Z_1

$$\begin{cases} Z_1 \sim \mathrm{NB}\left(\frac{\pi_2}{q_0}, N_2 - X'\right) \\ X' \sim \mathrm{NB}_{\mathrm{trunc}(N_2)}(p_1^{(1)}, N_1) \end{cases} \tag{S17}$$

and for Z_2

$$\begin{cases} Z_2 \sim \mathrm{NB}\left(\frac{\pi_1}{q_0}, N_1 - Y'\right) \\ Y' \sim \mathrm{NB}_{\mathrm{trunc}(N_1)}(1 - p_1^{(1)}, N_2) \end{cases} \tag{S18}$$

with the truncated negative binomial distribution $\mathrm{NB}_{\mathrm{trunc}(N)}(\cdot, \cdot)$ with the support $[0, N)$ ($N \in \mathbb{N}$). Based on these considerations, one can derive the expected number of patients with a positive test result for at least one biomarker during the time segment where only one subtrial is running. In case of subtrial 1 already closed, this number is given by

$$\begin{aligned} \mathrm{E}_1^{(2)} :&= \mathrm{E}[Z_1] \\ &= \frac{1}{\mathrm{P}[X < N_2]} \sum_{x=0}^{N_2-1} \frac{N_2 - x}{\frac{\pi_2}{q_0}} \binom{x + N_1 - 1}{x} \left(p_1^{(1)}\right)^{N_1} \left(1 - p_1^{(1)}\right)^x \\ &= \frac{1}{\mathrm{P}[X < N_2]} \left(N_2 \frac{q_0}{\pi_2} \sum_{x=0}^{N_2-1} \binom{x + N_1 - 1}{x} \left(p_1^{(1)}\right)^{N_1} \left(1 - p_1^{(1)}\right)^x \right. \\ &\quad \left. - \frac{q_0}{\pi_2} \sum_{x=0}^{N_2-1} x \binom{x + N_1 - 1}{x} \left(p_1^{(1)}\right)^{N_1} \left(1 - p_1^{(1)}\right)^x \right) \\ &= \frac{1}{\mathrm{P}[X < N_2]} \left(N_2 \frac{q_0}{\pi_2} \mathrm{P}[X < N_2] - \frac{q_0}{\pi_2} \mathrm{E}[X \, 1_{X < N_2}] \right) \\ &= \frac{q_0}{\pi_2} \left(N_2 - \mathrm{E}[X | X < N_2] \right) \\ &\overset{(S14)}{=} \frac{q_0}{\pi_2} \left(N_2 - \frac{1 - p_1^{(1)}}{p_1^{(1)}} \frac{\mathrm{I}_{p_1^{(1)}}(N_1 + 1, N_2 - 1)}{\mathrm{I}_{p_1^{(1)}}(N_1, N_2)} N_1 \right) \end{aligned} \tag{S19}$$

with the application of properties of the (truncated) negative binomial distribution. Analogously in case of subtrial 2 already closed, the expected number of patients with a positive test result for at least one biomarker during the time segment where only subtrial 1 is running is given by

$$\begin{aligned} \mathrm{E}_2^{(2)} :&= \frac{q_0}{\pi_1} \left(N_1 - \mathrm{E}[Y | Y < N_1] \right) \\ &\overset{(S16)}{=} \frac{q_0}{\pi_1} \left(N_1 - \frac{p_1^{(1)}}{1 - p_1^{(1)}} \frac{\mathrm{I}_{1-p_1^{(1)}}(N_2 + 1, N_1 - 1)}{\mathrm{I}_{1-p_1^{(1)}}(N_2, N_1)} N_2 \right) \end{aligned} \tag{S20}$$

Finally, let N_{screen} denote the total number of patients needed to be screened to

complete the considered umbrella trial. Then, $q_0\,\mathrm{E}[N_{\mathrm{screen}}]$ corresponds to the expected number of patients with a positive test result for at least one biomarker during the complete trial. Hence, combining the previous results leads to

$$\mathrm{E}[N_{\mathrm{screen}}] = \left\{ \left(\mathrm{E}_1^{(1)} + \mathrm{E}_1^{(2)}\right) \mathrm{P}[X < N_2] + \left(\mathrm{E}_2^{(1)} + \mathrm{E}_2^{(2)}\right) \mathrm{P}[Y < N_1] \right\} \frac{1}{q_0} \tag{S21}$$

Biomarker status distribution. Consider the time segment with both subtrials running, first. In this period, let $n^{(1)}_{i,\left(\mathrm{B}_1^+,\mathrm{B}_2^+\right)}$ denote the expected number of patients with biomarker status $\left(\mathrm{B}_1^+,\mathrm{B}_2^+\right)$ in subtrial i. Then, it holds

$$\begin{aligned}
n^{(1)}_{1,(1,0)} &= \frac{\mathrm{P}[(1,0)]}{p^{(1)}_{0,1}} \left(N_1\,\mathrm{P}[X < N_2] + \mathrm{E}[Y|Y < N_1]\,\mathrm{P}[Y < N_1]\right) \\
n^{(1)}_{1,(1,1)} &= \frac{r\,\mathrm{P}[(1,1)]}{p^{(1)}_{0,1}} \left(N_1\,\mathrm{P}[X < N_2] + \mathrm{E}[Y|Y < N_1]\,\mathrm{P}[Y < N_1]\right) \\
n^{(1)}_{2,(0,1)} &= \frac{\mathrm{P}[(0,1)]}{p^{(1)}_{0,2}} \left(N_2\,\mathrm{P}[Y < N_1] + \mathrm{E}[X|X < N_2]\,\mathrm{P}[X < N_2]\right) \\
n^{(1)}_{2,(1,1)} &= \frac{(1-r)\,\mathrm{P}[(1,1)]}{p^{(1)}_{0,2}} \left(N_2\,\mathrm{P}[Y < N_1] + \mathrm{E}[X|X < N_2]\,\mathrm{P}[X < N_2]\right)
\end{aligned} \tag{S22}$$

Now, consider the time segment where one subtrial is already closed. In this period, let $n^{(2)}_{i,\left(\mathrm{B}_1^+,\mathrm{B}_2^+\right)}$ denote the expected number of patients with biomarker status $\left(\mathrm{B}_1^+,\mathrm{B}_2^+\right)$ in subtrial i in case subtrial k is already closed ($k = 1, 2,\ k \neq i$). Then,

$$\begin{aligned}
n^{(2)}_{1,(1,0)} &= \frac{\mathrm{P}[(1,0)]}{q_0}\,\mathrm{E}_2^{(2)}\,\mathrm{P}[Y < N_1] \\
n^{(2)}_{1,(1,1)} &= \frac{\mathrm{P}[(1,1)]}{q_0}\,\mathrm{E}_2^{(2)}\,\mathrm{P}[Y < N_1] \\
n^{(2)}_{2,(0,1)} &= \frac{\mathrm{P}[(0,1)]}{q_0}\,\mathrm{E}_1^{(2)}\,\mathrm{P}[X < N_2] \\
n^{(2)}_{2,(1,1)} &= \frac{\mathrm{P}[(1,1)]}{q_0}\,\mathrm{E}_1^{(2)}\,\mathrm{P}[X < N_2]
\end{aligned} \tag{S23}$$

Finally, the expected biomarker status distribution in a subtrial corresponds to the conditional probabilities of having a specific biomarker status given the considered subtrial. Hence, the results for the two time segments provided in equations (S22) and (S23) must be added and the sums divided by the respective subtrial sizes, i.e.

$$\mathrm{P}\left[\left(\mathrm{B}_1^+,\mathrm{B}_2^+\right)\,|\,\text{subtrial } i\right] = \frac{n^{(1)}_{i,\left(\mathrm{B}_1^+,\mathrm{B}_2^+\right)} + n^{(2)}_{i,\left(\mathrm{B}_1^+,\mathrm{B}_2^+\right)}}{N_i}$$

Thus, the biomarker status distribution is given by

$$
\begin{aligned}
&\mathrm{P}[(1,0)\,|\,\text{subtrial 1}] \\
&\quad = \frac{\mathrm{P}[(1,0)]}{N_1} \left\{ \frac{N_1}{p_{0,1}^{(1)}} \, \mathrm{P}[X < N_2] + \left(\frac{\mathrm{E}[Y|Y < N_1]}{p_{0,1}^{(1)}} + \frac{\mathrm{E}_2^{(2)}}{q_0} \right) \mathrm{P}[Y < N_1] \right\} \\
&\mathrm{P}[(1,1)\,|\,\text{subtrial 1}] \\
&\quad = \frac{\mathrm{P}[(1,1)]}{N_1} \left\{ \frac{r\,N_1}{p_{0,1}^{(1)}} \, \mathrm{P}[X < N_2] + \left(\frac{r\,\mathrm{E}[Y|Y < N_1]}{p_{0,1}^{(1)}} + \frac{\mathrm{E}_2^{(2)}}{q_0} \right) \mathrm{P}[Y < N_1] \right\} \\
&\mathrm{P}[(0,1)\,|\,\text{subtrial 2}] \\
&\quad = \frac{\mathrm{P}[(0,1)]}{N_2} \left\{ \frac{N_2}{p_{0,2}^{(1)}} \, \mathrm{P}[Y < N_1] + \left(\frac{\mathrm{E}[X|X < N_2]}{p_{0,2}^{(1)}} + \frac{\mathrm{E}_1^{(2)}}{q_0} \right) \mathrm{P}[X < N_2] \right\} \\
&\mathrm{P}[(1,1)\,|\,\text{subtrial 2}] \\
&\quad = \frac{\mathrm{P}[(1,1)]}{N_2} \left\{ \frac{(1-r)\,N_2}{p_{0,2}^{(1)}} \, \mathrm{P}[Y < N_1] + \left(\frac{(1-r)\,\mathrm{E}[X|X < N_2]}{p_{0,2}^{(1)}} + \frac{\mathrm{E}_1^{(2)}}{q_0} \right) \mathrm{P}[X < N_2] \right\}
\end{aligned}
\tag{S24}
$$
