# Supplementary material for: Effect size estimates from umbrella designs: Handling patients with a positive test result for multiple biomarkers using random or pragmatic subtrial allocation
Source: PLoS One. 2020 Aug 14;15(8):e0237441. doi: 10.1371/journal.pone.0237441 (PMC7428134; doi:10.1371/journal.pone.0237441)
Supplement: S2 Note — (PDF) [file pone.0237441.s003.pdf]

S2 Note Weighted linear regression as (sub-) trial analysis method

Settings and notations. Suppose that we have two experimental treatments related to two different, binary biomarkers B_i ($i = 1, 2$). We denote a patient's combined test results for all biomarkers the patient's biomarker status. Let B_i^+ be an indicator for a positive test result for B_i. Then, let the tuple $(\mathrm{B}_1^+, \mathrm{B}_2^+)$ denote the biomarker (positive) status of a patient. We consider two independent parallel group trials and the corresponding umbrella trial with two parallel group subtrials. The size of (sub-) trial i is N_i. For further details on the designs we refer to the Section "Investigated study design scenarios".

Weighted linear regression model. Consider (sub-) trial i. Let $X_{j,i}$ indicate whether patient j receives the experimental treatment and $Y_{j,i}$ be the outcome of patient j. Let $\beta_{0,i}$ and $\beta_{1,i}$ denote the regression coefficients that are estimated by minimising

$$\sum_{j=1}^{\mathrm{N}_i} w_{j,i} \left(Y_{j,i} - \beta_{0,i} - \beta_{1,i}\, X_{j,i}\right)^2$$

for given weights $w_{j,i}$. Though, $\beta_{1,i}$ represents the treatment effect estimate. The weight

for patient j in subtrial i with the biomarker status $(\mathrm{B}_1^+, \mathrm{B}_2^+)$ is given by

$$w_{j,i} = \frac{\mathrm{P}[(\mathrm{B}_1^+, \mathrm{B}_2^+) \,|\, \text{trial } i]}{\mathrm{P}[(\mathrm{B}_1^+, \mathrm{B}_2^+) \,|\, \text{subtrial } i]} \tag{S25}$$

The conditional probability for exhibiting the biomarker status $(\mathrm{B}_1^+, \mathrm{B}_2^+)$ in (sub-) trial i ($\mathrm{P}[(\mathrm{B}_1^+, \mathrm{B}_2^+) \,|\, \text{(sub-) trial } i]$) is given in equations (10) and (12). These weights artificially mimic the biomarker status distribution of the independent trial i and, consequently, the weights are all equal to 1 for trial i.

Calculation of the weights in practice. In case of unknown true biomarker status distributions in the (sub-) trials, the estimated proportion of a given biomarker status $(\mathrm{B}_1^+, \mathrm{B}_2^+)$ in trial i can be derived from the given umbrella trial as follows: (1) Consider all subtrials in which patients with the biomarker status $(\mathrm{B}_1^+, \mathrm{B}_2^+)$ are included. (2) Select all patients in these subtrials that would have been eligible for trial i. (3) Order these patients by increasing inclusion time. (4) Take the proportion of patients with the biomarker status $(\mathrm{B}_1^+, \mathrm{B}_2^+)$ among the first N_i selected patients. If there are more than two biomarkers, e.g. m, perform steps (1) to (4) for the given biomarker status $(\mathrm{B}_1^+, \dots, \mathrm{B}_m^+)$ and the corresponding subtrials. Note that these weights are approximations, only.
