# Supplementary material for: Effect size estimates from umbrella designs: Handling patients with a positive test result for multiple biomarkers using random or pragmatic subtrial allocation
Source: PLoS One. 2020 Aug 14;15(8):e0237441. doi: 10.1371/journal.pone.0237441 (PMC7428134; doi:10.1371/journal.pone.0237441)
Supplement: S3 Note — (PDF) [file pone.0237441.s004.pdf]

S3 Note Additional results from the real data application
