# Supplementary figures and images for: Effect size estimates from umbrella designs: Handling patients with a positive test result for multiple biomarkers using random or pragmatic subtrial allocation
Source: PLoS One. 2020 Aug 14;15(8):e0237441. doi: 10.1371/journal.pone.0237441 (PMC7428134; doi:10.1371/journal.pone.0237441)

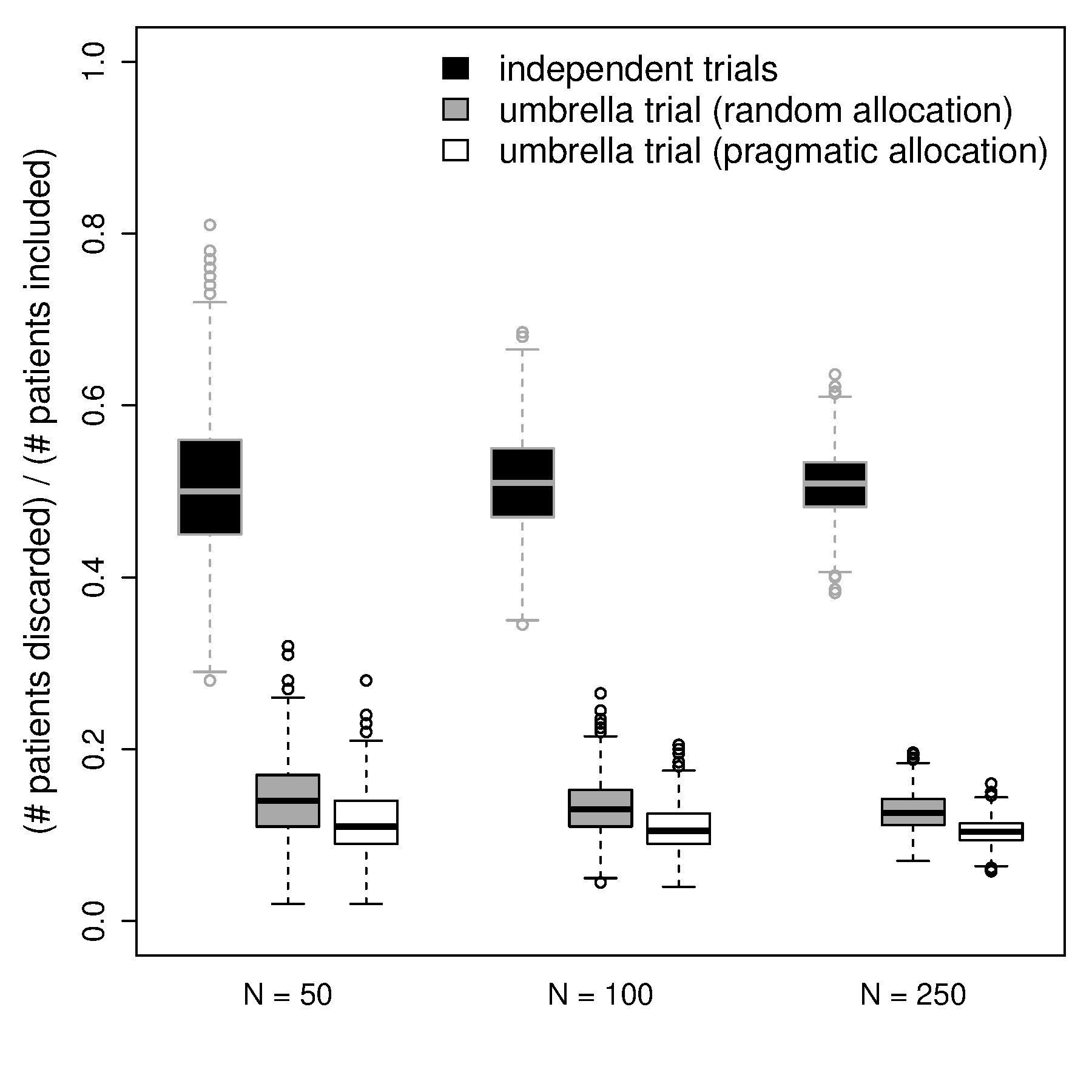

Supplement: S1 Fig — The trial size N is indicated. A “discarded patient” is a patient that was screened but not included in a (sub-) trial. The ratio is derived as Nscreen−2N divided by 2N. Nscreen denotes the number of screened patients in a bootstrap run. There are 2N included patients in each design. The distribution across the 1, 000 bootstrap runs are provided. (TIF) [file pone.0237441.s005.tif]

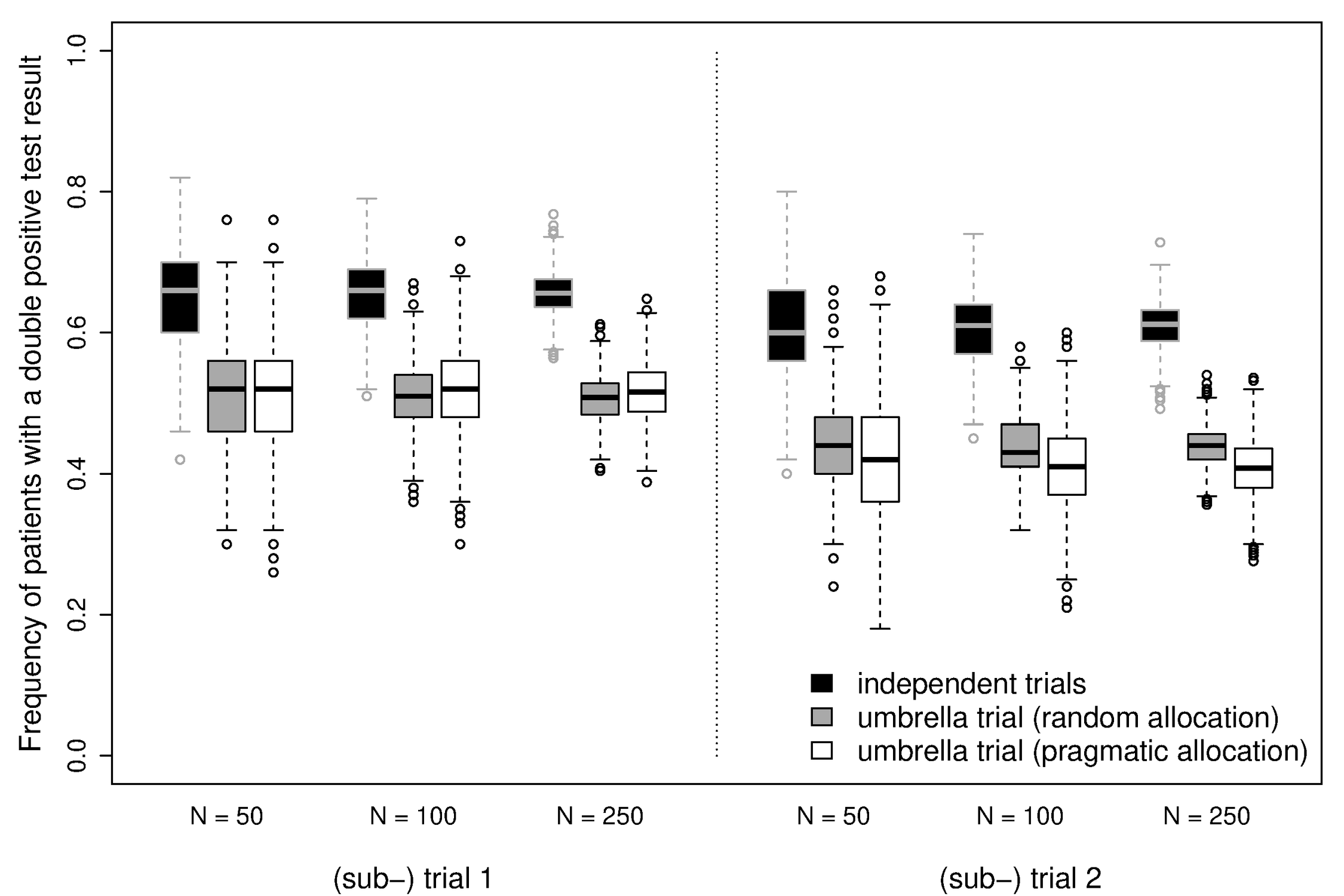

Supplement: S2 Fig — The trial size N and the respective subtrial are indicated. The distribution across the 1, 000 bootstrap runs are provided. (TIF) [file pone.0237441.s006.tif]
