# Supplementary material for: Effect size estimates from umbrella designs: Handling patients with a positive test result for multiple biomarkers using random or pragmatic subtrial allocation
Source: PLoS One. 2020 Aug 14;15(8):e0237441. doi: 10.1371/journal.pone.0237441 (PMC7428134; doi:10.1371/journal.pone.0237441)
Supplement: S1 Table — (PDF) [file pone.0237441.s007.pdf]

S1 **Table. Results from five exemplary (bootstrap) samples in the real data application for the independent trial design and both umbrella designs.**

Run	Independent trial design		Umbrella design (random allocation)		Umbrella design (pragmatic allocation)	
	trial 1	trial 2	subtrial 1	subtrial 2	subtrial 1	subtrial 2
Ratio of discarded to included patients						
1	0.43		0.09		0.04	
2	0.50		0.11		0.09	
3	0.59		0.12		0.12	
4	0.49		0.08		0.07	
5	0.60		0.20		0.21	
Proportion of patients with a double positive test results						
1	0.72	0.62	0.54	0.38	0.58	0.30
2	0.66	0.70	0.48	0.56	0.44	0.58
3	0.58	0.62	0.46	0.48	0.46	0.48
4	0.62	0.54	0.50	0.46	0.50	0.44
5	0.70	0.58	0.56	0.46	0.56	0.44
Weights						
. . . for patients with a single positive test result						
1	1.00	1.00	0.61	0.61	0.67	0.54
2	1.00	1.00	0.65	0.68	0.61	0.71
3	1.00	1.00	0.78	0.73	0.78	0.73
4	1.00	1.00	0.76	0.85	0.76	0.82
5	1.00	1.00	0.68	0.78	0.68	0.75
. . . for patients with a double positive test result						
1	1.00	1.00	1.33	1.63	1.24	2.07
2	1.00	1.00	1.38	1.25	1.50	1.21
3	1.00	1.00	1.26	1.29	1.26	1.29
4	1.00	1.00	1.24	1.17	1.24	1.23
5	1.00	1.00	1.25	1.26	1.25	1.32
Estimated treatment effect						
un-weighted						
1	1.26	−0.23	1.78	−0.36	1.33	−0.11
2	0.55	−0.35	0.83	−0.10	1.24	−0.35
3	−0.04	−0.65	0.81	0.53	1.70	−0.37
4	−1.67	−1.94	−1.37	−0.57	−1.40	−0.32
5	0.03	−0.34	0.65	−0.96	0.59	−0.52
weighted						
1	–	–	1.30	−0.80	0.74	−0.18
2	–	–	0.46	0.00	0.89	−0.36
3	–	–	−0.01	0.41	1.11	−0.74
4	–	–	−1.61	−0.74	−1.64	−0.55
5	–	–	0.09	−1.18	0.09	−0.71

The (sub-) trial size is 50. Each sample is consecutively numbered (run). The ratio of the number of discarded patients to the number of included patients can only be provided for complete trials. A “discarded patient” is a patient that was screened but not included in a (sub-) trial. The ratio is derived as the difference between the number of screened patients and the number of included patients. The number of included patients is 100. Weighted linear regression is not applicable in the independent trial design as all weights are 1.
