# Supplementary material for: Effect size estimates from umbrella designs: Handling patients with a positive test result for multiple biomarkers using random or pragmatic subtrial allocation
Source: PLoS One. 2020 Aug 14;15(8):e0237441. doi: 10.1371/journal.pone.0237441 (PMC7428134; doi:10.1371/journal.pone.0237441)
Supplement: S2 Table — (PDF) [file pone.0237441.s008.pdf]

S2 **Table. Application of the analytical calculation formulae to the real data and its comparison with the bootstrap means for the independent trial design and the umbrella design with the random allocation scheme.**

(a) Number of discarded (screened but not included) patients.

(Sub-) trial size	Analytical expectation	Bootstrap mean
Independent trial design		
50	51.0	51.0
100	102.1	102.3
250	255.2	254.1
Umbrella design (random allocation) – with $\phi = -0.09$		
50	14.1	14.1
100	26.8	26.6
250	64.3	63.6
Umbrella design (random allocation) – with $\phi = 0$		
50	16.4	–
100	31.5	–
250	76.2	–

(b) Proportion of patients with a positive test result for both biomarkers.

(Sub-) trial size	Analytical expectation		Bootstrap mean	
	(sub-) trial 1	(sub-) trial 2	(sub-) trial 1	(sub-) trial 2
Independent trial design				
50	0.69	0.64	0.65	0.61
100	0.69	0.64	0.66	0.61
250	0.69	0.64	0.66	0.61
Umbrella design (random allocation) – with $\phi = -0.09$				
50	0.51	0.44	0.51	0.44
100	0.51	0.44	0.51	0.44
250	0.51	0.44	0.51	0.44
Umbrella design (random allocation) – with $\phi = 0$				
50	0.55	0.47	–	–
100	0.54	0.47	–	–
250	0.54	0.47	–	–

(c) Difference (ΔX_i) between the treatment effect estimated in an umbrella trial and the (true) effect δ estimated in an independent trial.

(Sub-) trial size	Analytical expectation		Bootstrap mean	
	subtrial 1	subtrial 2	subtrial 1	subtrial 2
Umbrella design (random allocation) – with $\phi = -0.09$				
50	0.22	0.07	0.26	0.08
100	0.23	0.08	0.25	0.09
250	0.23	0.08	0.30	0.08
Umbrella design (random allocation) – with $\phi = 0$				
50	0.24	0.08	–	–
100	0.24	0.08	–	–
250	0.25	0.08	–	–

The analytical expectations are given in the Section "Impact of the umbrella design with the random allocation scheme" and the definition of the bootstrap means in the Section "Impact of the pragmatic subtrial allocation scheme and the analysis method". Underlying reduced data set characteristics relied on for the expectations are the prevalence of the biomarkers related to the (sub-) trials ($\pi_1 = 0.638$, $\pi_2 = 0.688$), the correlation (dependency) of the biomarkers ($\phi = -0.09$) and the estimated true treatment effect ($\delta = 0.3539$). Additionally for the analytical calculations for the umbrella design, independent biomarkers ($\phi = 0$) are assumed.
