# Supplementary material for: Effect size estimates from umbrella designs: Handling patients with a positive test result for multiple biomarkers using random or pragmatic subtrial allocation
Source: PLoS One. 2020 Aug 14;15(8):e0237441. doi: 10.1371/journal.pone.0237441 (PMC7428134; doi:10.1371/journal.pone.0237441)
Supplement: Attachment — Submitted filename: Kesselmeier_et_al_Response_to_Reviewer_to_PONE-D-18-36382R1.pdf [file pone.0237441.s009.pdf]

Reviewer #1

After having read the second version of the manuscript I am still not fully convinced of the way the research is presented.

RESPONSE: Thank you for your thorough review. Please note that Chapter 3 changed as there were errors in the analytical deductions. Furthermore, the real data application was adapted to get more informative results.

1) It seems that the authors have a different understanding of basket and umbrella trials. It is acknowledged that sometimes different definitions are used. However, these more often are related to the term "platform trial" then to "basket trial" or "umbrella trial".
Woodcock and LaVange define these trials in a recent publication as follows:

Umbrella: To study multiple targeted therapies in the context of a single disease

Basket: To study a single targeted therapy in the context of multiple diseases or disease subtypes

In the main text it seems that the authors adapt this definition of biomarker driven targeted therapies, were the biomarker of interest is often (but not necessarily) considered the molecular target of the treatment. However, in the discussion the authors write:

> "Another master protocol design is the basket design, e.g. [1, 2]. In a basket design, one biomarker-driven experimental treatment is investigated for several diseases / molecular targets - e.g. a particular receptor which has an impact for both breast and ovarian cancer. Thus, each disease/target defines a subtrial. Consequently, our observations for the umbrella design should also translate to basket trials by switching the role of biomarker and disease/target when defining the subtrials - although patients exhibiting more than one target are probably rarer than patients with positive test results for more than one biomarker and might not be included in a basket trial at all."

It is acknowledged that one could of course study one drug for multiple biomarker defined subgroups. This is usually the case in standard developments where a single indication with multiple biomarker defined subgroups is studied on an "all comer" basis. However, this would hardly be called a basket trial. Having different targets but the same targeted therapy does not seem to be a sensible way forward as we would then have a mismatch of target and therapy. The usual connotation of basket trial refers to multiple "classical" diseases / tumor locations which all express the same target and which are studied in one histology independent trial. In this situation, it is then the number of diseases / tumor locations which would have to be co-expressed. While this could happen, I assume (I am no clinician though) that one would most likely have a primary tumor (type) which is to be treated. The citation of Simon (2017) does not fit at all to clarify the statement of the authors as it simply explains the basic concept of a molecular target, which is the basis of both basket and umbrella trials.

RESPONSE: We rewrote the discussion focussing on more clarity regarding terms like „target".

2) The pragmatic allocation is only discussed in comparison to the random allocation in the simulation studies. The authors should improve the discussion of the pragmatic approach by including it in the theoretical derivation and discussing it separately.

RESPONSE: We clarified the presentation. We highlight that the observation from our first objective was based on the random allocation scheme, only. Then, we extended the presentation of results of the simulation study. We now provide comparisons between the two umbrella designs and with the

independent trial design. Furthermore, we discuss them together in Section 5. Please note that we corrected the analytical derivations for the random allocation scheme.

3) The new data example is not very instructive.

a. They only have one subtrial (it seems they try to mimic the first subtrial which was assumed to recruit faster). I also cannot fully follow the ideas of the authors: It seems that they only have one biomarker (lactate level) but declare this biomarker to constitute two biomarkers (if this biomarker is expressed it is assumed that BM results are double positive). That is completely odd.

b. The results for the recruitment and biomarker distribution are trivial and were discussed before. Regarding the much more interesting treatment effect estimate(s) it seems that the proposed weighted regression approach does not work. Effect estimates without weights are overall closer to the "independent trial" than with weighting.

c. No comparison between (BM1, BM2) = (1,0) and (1,1) patients was presented. However, this would allow a deeper understanding of the impact of the lactate biomarker on treatment effects. As already stated in the first review, these subgroup analyses would be considered very relevant as they are not affected by the BM distribution.

RESPONSE:

a. We agree that the underlying approach was not presented well. However, we adapted the real data application. Now, we use the data of a randomised controlled trial (RCT) as population from which we draw bootstrap samples. This approach allows building complete umbrella trials (with two subtrials) and the corresponding independent trials. Therefore, we defined to binary biomarkers. Please note that there is no biomarker at all in the original RCT. We hope that this approach is less odd but it is still to some extent arbitrarily. For a real design comparison, an umbrella design and the corresponding independent trial design must be conducted in parallel which is probably unethical and logistically rather impossible.

b. The observed treatment effects were an additional reason to rebuild the real data application. Now, the results are more informative. This also applies to the effect size estimates.

c. If we understand the reviewer correct, the reviewer proposed a subgroup analysis or the extension of the linear regression model by the patient's biomarker status (and the interaction with the treatment). As these analyses are design-driven and probably not biology-driven we would consider them as sensitivity analysis, only (see Section 5 for a discussion of this issue).

4) Please note that Student's t-test is not an estimate (but a test). The corresponding estimate would be the mean difference. On the other hand, weighted linear regression is a method and thus neither an estimate nor a test (per se). However, effect estimates (based on regression coefficients) and tests (also based on some statistic of the regression coefficients) can be derived based on the model. This should be clarified and rephrased in the manuscript.

RESPONSE: We clarified this aspect in the text as well as in the figures. Thank you.

<u>Reviewer #2</u>

The authors addressed in their revised version some of my comments but not sufficiently my main ones. I am convinced that the topic has some potential for an interesting paper on relevant and topical questions related to umbrella designs. However, I still have some major concerns that were not sufficiently addressed, see below, in addition to some minor suggestions, and believe that the

paper would need some more profound investigations as suggested in my comments, to be eligible for publication.

RESPONSE: Thank you for your thorough review and the detailed remarks. Please note that Chapter 3 changed as there were errors in the analytical deductions. Furthermore, the real data application was adapted to get more informative results.

1. The analytical derivations to calculate the expected number of patients to be screened as well as the biomarker status (section 3.1.and 3.2) are still incorrect. From Note S2 (but not from section 3.1 itself) it becomes clear that in Formula (2) the subtrial to close first should be determined by the subtrial with the lower expected value of the number of subjects to be screened in the first part. However, the subtrial to close first is a random variable with a Bernoulli distribution, which has to be considered correctly in the derivation of expectation of the total number of subjects to be screened. Actually, the probability that subtrial 1 closes first can be expressed by the incomplete beta function. Also, the expected number of screened patients in the second part depends on a random number of patients still to be screened to the other subtrial after the first part, involving a mixture of two (negative binomial) distributions. Consequently, all derivations starting from (A4), line 537, are wrong. Obviously, if the probability of one subtrial to close first is very low or very high the given formula comes close to the correct expectation but the formula as stated remains incorrect and the expectation should be derived correctly (especially because it is perfectly possible). The authors state in their response that the simulations would confirm the analytical derivation but this is not true when considering, e.g., the example given by the authors with □1=0.24, □2=0.25, N1 = N2=100, where Formula (2) gives 473.5, but even the simulations from the authors using their R program with 10 000 simulation runs gives approximately 486 (286 + N1 + N2) as the median and also the mean when adapting the program code (by the way, I don't understand why the authors intend to confirm an expected value by a simulated median and not by the mean as it would be correct, but the difference is negligible). The exact number can actually be calculated using an exact formula to be 486.2, which I could also confirm by my one simulations with 100 000 runs. Also, the calculations of the biomarker distribution contain the same error and should be corrected. Of course, correct derivations are much more involved than those given by the authors, for both, the expected numbers of patients and the biomarker distribution, but appear to be perfectly possible.

RESPONSE: We agree and corrected the calculations. Thank you very much for your more detailed information on this issue. Furthermore, we adapted the simulation study so that the mean (instead of the median) is used, now.

2. As stated in my first review, the weights to be used in the weighted approach are not fixed but random numbers depending on the screening process. The properties and the validity of the weighted regression are usually based on weights that are fixed in advance. In any case, but especially in case the proportion of the double positive subgroup or the number of subjects is small, the variance of the estimate is increased and the distribution used to derive the statistical test or the confidence interval would change. This may not be an issue asymptotically when sample sizes are large. The authors added a simple statement that weights might be questionable if there are too few patients, but I would consider such a vague statement to be insufficient. Instead, the authors could evaluate the type-1 error of the procedure in which the empirical proportions are just plugged-in in the considered scenarios and present the asymptotic properties. If the type-1 error is violated, it would not be adequate to compare a design that maintains type-1 error with one that does not. Furthermore, using the expected proportions of the trial's biomarker distribution (if correctly

calculated and if the underlying 1 and 2 are considered to be known) to derive fixed weights would be an alternative option to discuss and to evaluate.

RESPONSE: Note that the weights are based on the given expected biomarker status distribution in the simulations study and on the observed distribution in the (sub-) trials in the real data application. We clarified this in the description. Regarding the effect size estimates, the aim of the manuscript is to point to the issue of differing effect size estimates between the trial designs. However, we do not want to leave the reader without any idea for a solution for the aspect of differing effect size estimates. In the revised version of the manuscript, we refer to research done related to the issues of a possible loss of efficiency.

3. The authors intend to derive the efficiency of the umbrella design as compared to the independent trials but base their conclusions on the number to be screened to obtain the sample size that lead to a specific power of the independent trials. However, what matters in terms of efficiency is the number to be screened to obtain the same power for each design. Certainly, there is no difference if the unweighted analysis is used, but for the weighted approach the sample size would have to be adjusted to obtain the same power. Again, a simple comment that weighting may affect power would be insufficient, if the intention is to properly compare the different designs. Instead, for the weighted approach the number of subjects to be screened should be derived such that the same power as in the independent trials is obtained. This should be a relatively simple exercise if the weights are fixed. If, however, the weights are determined from the empirical proportions this would be more difficult considering the distribution of empirical proportions and the potential power change when empirical proportions are plugged in.

RESPONSE: We intend to derive the efficiency gain when applying an umbrella design compared to the independent trial design and the price to be paid if the impact of the design choice is not acknowledged. We provide the weighted regression approach because we do not want to leave the reader without any idea of how to account for the design impact on the effect size estimates. Accounting for the impact of the weighting is part of future trial planning. Any illustration of the magnitude of an increase in the required sample size would be arbitrarily.

4. Although I appreciate a real data example, I fear that the presented semi-synthetic data sets appear to be - in the presented form - of “semi-interest”, I would say, since only one “subtrial” is considered and the typical umbrella design with several subtrials is not depicted. Wouldn’t it be possible to use two different baseline variables to mimic the setting of the umbrella design that is described in the paper? It could also show how the approaches behave and compare with a potentially small number of subjects in the double positive subgroup.

RESPONSE: We adapted the real data application. Now, we use the data of a randomised controlled trial (RCT) as population from which we draw bootstrap samples. This approach allows building complete umbrella trials (with two subtrials) and the corresponding independent trials. Therefore, we defined to binary biomarkers. Please note that there is no biomarker at all in the original RCT.

5. (Minor) I appreciate a discussion of correlated biomarkers since correlation appear to be more realistic. Although dependence may be described in different ways I would rather use the proper correlation = (P(1,1) - 12)/sqrt(1(1- 1)2(1- 2)) and I would suggest to already write the main body of the paper in a more general form without restriction to independent biomarkers, since this

can be done in a relatively simple way, just using as an additional fixed parameter, which could be set to 0 in some of the examples.

RESPONSE: We followed the suggestion and write the main body of the paper in a more general form without restriction to independent biomarkers. In the illustrative example and in the simulation study, however, we still assume independence. For the real data application, we provide information on the biomarker dependence.

6. (Minor) Whether and when it is useful to use the weighted approach, considering, e.g., a setting where effect differences are expected and one may advocate to use a stratified analysis only, may be a matter of debate, but it is certainly valid and useful to present, evaluate and discuss the approach to estimate the overall effect in the population of interest and the authors already presented some discussion on this point. I guess, however, that the discussion could be somewhat enhanced by discussing, e.g., the external validity of the estimate of the overall effect that is improved by the weighted approach.

RESPONSE: Done. Thank you.

7. (Minor) Notation should be checked for consistency. E.g. the use of both, Bi and Bi+, still appears to be confusing.

RESPONSE: We checked the notation. Bi is used if we consider the biomarker itself and Bi+ if we consider the test result for biomarker Bi.

<u>Reviewer #3</u>

The manuscript „Umbrella designs: handling patients with a positive test result for multiple biomarkers using random or pragmatic subtrial allocation” has improved and the revised manuscript incorporates most of the suggestions raised by the reviewers. However, there are still some things unclear:

RESPONSE: Thank you for your thorough review. Please note that Chapter 3 changed as there were errors in the analytical deductions. Furthermore, the real data application was adapted to get more informative results.

.) The notation in Table 1 is still confusing. E.g. E[N_screen,i,2nd] in the legend of Table 1 is not always 0 as stated in S2 (A6) because it is different for i=1,2. It may help to denote the subtrial that closes earlier w.l.o.g with subscript 1 and the other one with subscript 2 throughout the text or at least in Table 1 to avoid subscripts i and j.

RESPONSE: We apologise for the confusion. Following the reviewer's suggestions, we rethought and tried to simplify our notation as far as it was possible. Please note that parts of Section 3 changed due to the correction of the analytical formulae. Furthermore, we incorporated the content of Table 1 into the text.

.) It should also be described in the legend of Table 1, that it shows P[(B_1+,B_2+)|subtrial i]

RESPONSE: Table 1 was removed and the content included into the text.

.) Supporting information S2, row 553: There is no E[N_screen,i,2nd] in equation (A5)

RESPONSE: S2 Note is completely changed due to the necessary corrections of the analytical formulae.

.) Formula (4): the first row should be denoted as DeltaX_1 and the second as DeltaX_2 to avoid misconceptions. This should also be applied to Figures 4 and 5.

RESPONSE: Done.

.) It is not clear how d(1,0),1 (or the other d values respectively) is related to μ(1,0),1,EXP.
d(1,0),1= μ(1,0),1,EXP-0.2 only in the case of a stratified randomization between treatment and control with strata biomarker status ((1,0) or (1,1)). If the randomization is not stratified, the number of (1,1)-patients in the treatment and control group may not be equal, at least for smaller sample sizes.

RESPONSE: We provide the underlying formula for the calculations and the relation in the revised version of the manuscript. Furthermore, the expected distribution of the biomarker status is equal in both treatment arms - and we base our analyses on expectations.

.) In accordance to reviewer 1, I think that in the simulation study the reverse effect of (1,1) patients in the two subtrials is a rather unrealistic scenario. In the explanation of the simulation study it should be stated in more detail, that the reverse effect of (1,1) patients was chosen to show a scenario, where the design has an impact on the estimated treatment effect.

RESPONSE: These assumptions also apply in the illustrative example in section 3.4. There, we extended the motivation for this choice in the revised version.

.) All Figures should be given in higher quality. Text and numbers in the Figures are difficult to read.

RESPONSE: We agree that the resolution in the submitted version was not optimal but this was induced by the upload-system. The original figures exhibit a better resolution.
